# Postpartum Breastfeeding Practices and Attitudes in Parents: A Randomized Study to Evaluate the Effects of Individual and Group Breastfeeding Education of Mothers and Fathers

**DOI:** 10.7759/cureus.44811

**Published:** 2023-09-06

**Authors:** Yeşim Yeşil, Hafize Öztürk Can

**Affiliations:** 1 Midwifery Department, Mardin Artuklu University Faculty of Health Sciences, Mardin, TUR; 2 Midwifery Department, Ege University Faculty of Health Sciences, Izmir, TUR

**Keywords:** paediatrics, pediatrics, feeding method, formula feed, breast milk, nutrition, children, newborn, self-efficacy, breastfeeding

## Abstract

Background

Antenatal breastfeeding training is defined as the provision of breastfeeding information during pregnancy, which can be given in various ways, such as individual training and group training. The inclusion of fathers in this educational approach is associated with the initiation of breastfeeding, exclusive breastfeeding and duration of breastfeeding. However, studies involving fathers are limited. This randomized controlled study aimed to compare the effects of individual and group training given to parents and those of normal hospital practices on mothers’ breastfeeding self-efficacy and fathers' attitudes toward breastfeeding.

Methods

The study was conducted randomly in a training and research hospital between March 2014 and September 2014 and included 180 people. Of them, 90 were prospective mothers who were in the third trimester of their pregnancy and were living with their husbands and received service from the obstetrics outpatient clinic of the hospital. The expecting mothers and their husbands were randomly assigned to three groups: individual training, group training and control group. After randomization, prospective mothers and fathers in all groups received training. In the first week, sixth week, and fourth month after delivery, the mothers’ breastfeeding self-efficacy and breastfeeding attitudes as well as the attitudes of the fathers' toward breastfeeding were evaluated.

Results

There were no differences between the groups in terms of variables such as age, education status, family type, breastfeeding education status, and mode of delivery. There were significant differences between the scores obtained from the Breastfeeding Self-Efficacy Scale and its subscales in all three groups during the postpartum period (p <0.05). The highest scale scores were obtained at the postpartum fourth month in the individual training and control groups and at the postpartum sixth week in the group training group. There were differences between the scores obtained by the mothers and fathers during the postpartum process from the Iowa Infant Feeding Attitude Scale (p<0.05).

Conclusion

The analysis of all the results indicates that breastfeeding education given to parents in the antenatal period increases their breastfeeding self-efficacy and provides them with a positive attitude toward breastfeeding. However, further research is needed to determine whether individual or group training contributes to the development of breastfeeding self-efficacy and attitudes.

## Introduction

Breastfeeding (BF) is not only the safest and healthiest way to feed a baby but also contributes to the improvement of short- and long-term health outcomes for both the mother and the baby [1]. Evidence has indicated that BF offers many nutritional, immune-protective, and emotional benefits both for babies and their mothers [1,2]. The World Health Organization (WHO) and the American Academy of Pediatrics (AAP) recommend exclusive breastfeeding (EBF) in the first six months of life and continuation of breastfeeding for at least two years [3,4]. If BF is achieved under optimal conditions, it is estimated that it could prevent 823,000 child deaths and 20,000 breast cancer deaths each year [5]. Despite the significant benefits of BF, unfortunately, only 41% of babies under six months of age are exclusively breastfed [6]. According to the 2018 Turkey Demographic and Health Surveys (TDHS) results on EBF in Turkey, the average duration of breastfeeding is 16.7 months and the rate of EBF in children under six months of age is 41%, which aligns with that reported by the WHO [7].

Mothers' choices of BF type during the first few weeks after birth can be influenced by several factors [8]. In the literature, factors affecting BF are generally stated as the mother’s sociodemographic characteristics, physical and mental state, social support, and self-efficacy [9-11]. Of these factors, maternal breastfeeding self-efficacy (BSE) is of particular importance [9]. Self-efficacy is defined as an emotional attitude, one of the strongest predictors of a range of behaviors, including BF [12]. One of the factors that affects mothers' BF experiences and duration of BF is the help and support provided by other people. Intrafamily support, in particular spousal support, has come to the forefront. As reported, the baby’s father has an important effect on the mother's BF decisions and behaviors [13]. According to several studies, there is some evidence indicating that fathers' knowledge about the benefits and management of BF may affect the initiation and maintenance of BF [14-19].

Systematic reviews have shown that BF education interventions significantly contribute to increasing both the duration and effectiveness of BF. Methods such as face-to-face interviews and telephone counseling have been found to be effective. Various researchers compared telephone counseling and the face-to-face interview method and found evidence that the latter was more effective in increasing BF rates [20-23]. In terms of education, group and individual training are among the most common educational methods used to encourage BF in the antenatal period [22]. In addition, those who review relevant studies agree that long-term interventions that start in the antenatal period and continue in the postpartum period can yield more positive results [20,22,23]. Although there are studies that demonstrate the effectiveness of education given to the mother to increase the effectiveness of BF, there is no educational intervention study given both to prospective mothers and fathers. Therefore, we considered that it would be beneficial to investigate the results of education given to mothers and fathers together.

## Materials and methods

The dependent variables of the study were individual and group trainings. The independent variables were BSE and IOWA Infant Feeding Attitude Scale (IIFAS) scores. The study hypotheses are as follows: (1) BF education method affects mothers’ BSE; (2) BF education method affects mothers’ BF attitude; (3) BF education method affects fathers’ BF attitude.

Design

This is a controlled intervention study aimed at investigating the effectiveness of education given to prospective mothers and fathers. The study sample included women and their husbands who presented to the pregnant outpatient clinic of an Education Research Hospital in Izmir, a province in the western part of Turkey, between March 01, 2014 and September 30, 2014 and met the inclusion criteria.

Participants

The study included women who were primiparous, literate, able to communicate and 18 years and older, did not have risky pregnancy, did not receive BF education in the antenatal period, and whose gestational age was ≥37 weeks, and their husbands. The sample size was calculated using the Number Cruncher Statistical System-Power Analysis and Sample Size (NCSS-PASS) 2005 software (NCSS, LLC, Kaysville, Utah) (α = 0.05 and power = 99%). After the calculations, each of the three groups included 21 mothers and 21 prospective fathers. However, considering the possibility of withdrawals and/or dropouts in the study, the number of mothers and fathers was increased by 10% in each group. Therefore, each of the individual training, group training, and control groups comprised 30 mothers and 30 fathers. Allocation of the groups to the interview dates was performed through randomization, which is shown in Table 1. Due to the limitations of the study, data were collected on four working days other than Monday.

**Table 1:** Randomization of the three groups

	1^st^ Week	2^nd^ Week	3^rd^ Week	4^th^ Week
Tuesday	Control group	Individual education group	Group education group	Control group
Wednesday	Individual education group	Group education group	Control group	Individual education group
Thursday	Group education group	Control group	Individual education group	Group education group
Friday	Control group	Individual education group	Group education group	Control group

Data collection

Post-discharge follow-up form: A separate questionnaire was prepared for each follow-up. During the first week, sixth week, and fourth month follow-ups, the fathers' support status was explored. Breastfeeding Self-Efficacy Scale (BSES): This scale, developed by Cindy-Lee Dennis (1999) to assess BSE, consists of two subscales and 33 items. The Cronbach’s alpha value of the scale developed by Cindy-Lee Dennis was 0.95 [24]. The validity and reliability study of the Turkish version of the BSES was performed by Eksioglu and Ceber. In Eksioglu and Ceber’s study, Cronbach’s alpha coefficient was 0.91 for the overall scale and 0.89 for its technique subscale [25]. Technique subscale: This subscale aimed at defining the recognition of specific principles required for successful BF and mothers’ breastfeeding skills.

Intrapersonal thoughts subscale: This subscale includes items questioning mothers’ attitudes and beliefs toward BF. The items of the BSES are rated on a 5-point Likert-type scale ranging from 1 to 5 (1: not at all confident, 2: not confident, 3: sometimes confident, 4: often confident, and 5: always confident). As the total score obtained from the scale increases, so does the mother’s BF competence. The lowest and highest possible scores to be obtained from the scale are 33 and 165, respectively. On average, it takes approximately fifteen minutes to answer the items on the scale.

In the present study, the BSE was administered to the participating mothers four times. While the first administration was in the antenatal period before the education, the other administrations were in the first week, sixth week, and fourth month after birth.

Iowa Infant Feeding Attitude Scale (IIFAS): The 17-item IIFAS was developed by De La Mora and Russell to assess mothers' attitudes toward BF. The Cronbach’s alpha value of the IIFAS was 0.86 [26]. The validity and reliability study of the Turkish version of the IIFAS was performed by Yesil et al. (2013). In Yesil et al.’s study, the Cronbach’s alpha coefficient was calculated as 0.71. However, the paper was revised for publication and was published in a national journal in 2016 [27].

The IIFAS is designed to predict both the duration of BF and the method chosen for the feeding of the infant. The responses given to the items of the IIFAS are rated on a 5-point Likert-type scale ranging from 1 (strongly disagree) to 5 (strongly agree). Nine of the items support formula feeding; therefore, these items are reverse scored. While the minimum possible score to be obtained from the IIFAS is 17, the maximum score is 85. Whereas a higher mean score indicates that the mother’s attitude toward BF is positive, a lower mean score indicates that the mother prefers formula feeding more. In the present study, the Cronbach’s alpha value of the IIFAS was calculated as 0.62. On average, it took approximately 10 minutes to answer the items in the IIFAS.

In the present study, the BSES was administered to the participating mothers and fathers four times. It was first administered in the antenatal period before the education. The other administrations were performed in the first week, sixth week and fourth month after birth.

Educational materials

The educational materials used in the present study were the BF handbook, BF education slides, cloth breast models and baby doll puppets.

BF guidelines, BF education slides: Among the content of the slides are the anatomy of the breast, the importance of BF, the benefits of breast milk, BF techniques, breast problems, breast care, and the importance of spousal support in BF. In the guidelines, the focus is mostly on messages given through BF images.

Cloth breast models and baby doll puppets: During the education, the prospective mothers and fathers were not only provided with verbal information but were also shown the correct latching position, correct- incorrect BF position, etc., using baby doll puppets and cloth breast models. The cloth breast models and baby doll puppets were prepared by the researcher.

Measurement tools administered to the mothers: The descriptive characteristics questionnaire was used to question the sociodemographic and BF characteristics of all the participating mothers regardless of whether they were given education or not in the antenatal period. For each follow-up, a separate post-discharge follow-up form was prepared. The maternal follow-up forms were used to determine the mothers’ BF status, whether they had cracked nipples and whether they received support for BF in the first week, sixth week and fourth month after birth.

Measurement tools administered to the fathers: All participating mothers’ husbands (regardless of whether they were given education or not in the antenatal period) were administered the descriptive characteristics questionnaire to question their sociodemographic characteristics and whether they provided support to encourage their wives to breastfeed.

Intervention

The prospective mothers and fathers in the individual training group were given one-on-one BF education. The prospective mothers and fathers in the group training group were given BF education in groups of five couples. The prospective mothers and fathers in the control group underwent a routine clinical protocol.

In the first week after birth, both the mothers and fathers were interviewed face-to-face in the hospital and their BF status was questioned. In the first week after discharge, the mothers were administered the follow-up form, BSES, and IIFAS, whereas the fathers were administered the follow-up form and IIFAS. At the six-week and four-month follow-ups after discharge, both fathers and mothers were contacted via telephone. During the process, the mothers were administered the follow-up form, BSES, and IIFAS, while the fathers were administered the follow-up form and IIFAS.

Individual education group

During the antenatal period, the mood of the prospective mothers and fathers who did not have BF experience was taken into consideration to better understand the education given. When they felt well, the education was provided with adult education principles in mind.

During the education, BF education slides, cloth breast models and baby doll puppets were used. BF education was given to them by first providing information, then exemplifying BF and finally having them practice BF. The content of the education included the anatomy of the breast, the importance of BF, the benefits of breast milk, BF techniques, breast problems, breast care, and the importance of spousal support in BF. The education lasted approximately 60 minutes. The interactive teaching technique was used during the education when the prospective mothers and fathers were allowed to walk around, eat, and drink. At the end of the education, they were allowed to ask questions if they had any, and then their feedback on the education was received.

Group training group

The prospective mothers and fathers in the group training group received BF education in groups. During the education, BF education slides, cloth breast models, and baby doll puppets were used. The BF education was given by first giving information, then exemplifying BF and finally having them practice BF. The content of the education included the anatomy of the breast, the importance of BF, the benefits of breast milk, BF techniques, breast problems, breast care, and the importance of spousal support in BF. The education lasted approximately 60 minutes. Before the education, the couples in the group were introduced to each other and provided their consent indicating that they agreed to receive the education in the group. In addition, they provided their consent indicating that they would not disclose what was shared in the educational environment to third parties in order to ensure the participants’ privacy. The interactive teaching technique was used during the education. During the education period, the prospective mothers and fathers were allowed to walk around, eat, and drink. At the end of the education, the participants were allowed to ask questions if they had any, and then their feedback on the education was received.

Control group

The prospective mothers and fathers in this group underwent no special interventions except for routine care procedures in the clinic. While the mothers were administered the follow-up form, BSES and IIFAS, the fathers were administered the follow-up form and IIFAS. They were followed up during the postpartum follow-ups, and when they asked for help, they were referred to those from whom they could receive help.

Data analysis

SPSS version 16.0 (SPSS Inc., Chicago, IL) was used for the statistical analysis of the data. Intergroup descriptive characteristics are presented with number-percentage distributions. The Kruskal-Wallis test was used to compare the mean scores of the prospective mothers and fathers obtained from the BSES and IIFAS during pregnancy and at the first week, sixth week, and fourth month follow-ups after birth. The K-related samples test was used for the intragroup comparisons of the mean BSES and IIFAS scores obtained during the follow-ups. A p-value of <0.05 indicated statistical significance.

Ethical approval

This prospective, randomized controlled trial was approved by Katip Çelebi University’s Ethics Committee (decision date: February 13, 2014, decision number: 18) and was registered as a clinicaltrials.Gov under identifier NCT04021667. All participants signed an informed consent form.

## Results

The results are given under three headings: 1. Data related to the sociodemographic characteristics, 2. Results obtained from the BSES, and 3. Results obtained from the IIFAS Scale. Table 2 shows the study results and the sociodemographic characteristics of the mothers and fathers.

**Table 2:** Characteristics of the study participants BF: Breastfeeding; C-section: Cesarean delivery

	Individual education group (n=30)	Group education group (n=30)	Control group (n=30)	p
	Mean ± SD	Mean ± SD	Mean ± SD	
Age (years)				
Maternal	25.8±7.4	23.76±4.74	23.66±3.95	0.242
Paternal	28.2±4.9	27.9±5.6	27.8±3.5	0.939
	Number (%)	Number (%)	Number (%)	
Maternal education				
Elementary school	8 (26.7)	8 (26.7)	11 (36.7)	0.630
Higher	22 (73.3)	22 (73.3)	19 (63.3)	
Paternal eduation				
Elementary school	4 (13.3)	7 (23.3)	8 (26.7)	0.429
Higher	26 (86.7)	23 (76.7)	22 (73.3)	
Social security				
Yes	28(93.3)	28(93.3)	27 (90.0)	0.861
No	2 (6.7)	2 (6.7)	3 (10.0)	
Family type				
Nuclear	23 (76.7)	20 (66.7)	20 (66.7)	0.630
Extended	7 (23.3)	10 (33.3)	10 (33.3)	
Previous BF education				
Yes	7 (23.3)	9 (30.0)	8 (26.7)	0.848
No	23 (76.7)	21 (70.0)	22 (73.3)	
Mode of delivery				
Vaginal	6 (20.0)	13 (44.8)	7 (23.3)	0.077
C-Section	23 (80.0)	16 (55.2)	23 (76.7)	

The study was completed with 90 mothers and 90 fathers. Table 1 shows the characteristics of the mothers and fathers in the individual training, group training, and control groups. There was no difference between the groups in terms of variables such as age, educational status, family type, BF education status, and mode of delivery. These results indicate that the prospective mothers and fathers were similar in terms of their sociodemographic characteristics. Table 3 presents the BF characteristics of the participating mothers in the first week, sixth week and fourth month after birth.

**Table 3:** Breastfeeding characteristics of the participating mothers at the first week, sixth week and fourth month after birth *As one infant died in the first week after birth in the group education group, the study was completed with 29 participants in this group EBF: Exclusive breastfeeding

	Individual education group			Group education group			Control group		
	1^st^ week	6^th^ week	4^th^ month	1^st^ week	6^th^ week	4^th^ month	1^st^ week	6^th^ week	4^th^ month
	N (%)	N (%)	N (%)	N (%)	N (%)	N (%)	N (%)	N (%)	N (%)
Feeding type									
EBF	21 (70.0)	28 (93.3)	19 (63.3)	25 (86.2)	24 (82.8)	14 (48.3)	19 (63.3)	11 (36.7)	4 (13.3)
Breast milk and formula									
EBF and liquids	-	-	6 (20.0)	1 (3.5)	4 (13.8)	11 (37.9)	5 (16.7)	15 (50.0)	18 (60.0)
EBF and formula	9 (30.0)	2 (6.7)	5 (16.7)	3 (10.3)	1 (3.4)	4 (13.8)	6 (20.0)	3 (10.0)	5 (16.7)
Formula	-	-	-	-	-	-	-	1 (3.3)	3 (10.0)
Reasons for giving food other than breast milk									
Inadequate milk supply	4 (44.4)	2 (100.0)	4 (36.4)	1 (33.3)	1 (20.0)	2 (13.3)	5 (83.3)	8 (42.1)	11 (42.3)
Others	5 (55.6)	-	7 (63.6)	2 (66.7)	4 (80.0)	13 (86.7)	1 (16.7)	11 (57.9)	15 (57.7)
Having a Breast Problem									
Yes	20 (66.7)	8 (26.7)	1 (3.3)	13 (44.8)	4 (13.8)	2 (6.9)	20 (66.7)	12 (40.0)	1 (3.3)
No	10 (33.3)	22 (73.3)	29 (96.7)	16 (55.2)	25 (86.2)	27 (93.1)	10 (33.3)	18 (60.0)	29 (96.7)
Total	30 (100.0)	30 (100.0)	30 (100.0)	29 (100.0)	29 (100.0)	29 (100.0)	30 (100.0)	30 (100.0)	30 (100.0)
Breast problem experienced**									
Breast engorgement	2 (10.0)	1 (12.5)	1 (100.0)	-	1 (25.0)	2 (100.0)	-	-	1 (100.0)
Cracked nipples	18 (90.0)	7 (87.5)	-	13 (100.0)	3 (75.0)	-	20 (100.0)	12 (100.0)	-
Total	20 (100.0)	8 (100.0)	1 (100.0)	13 (100.0)	4 (100.0)	2 (100.0)	20 (100.0)	12 (100.0)	1 (100.0)

At the first week of follow-up after birth, the highest EBF rate was in the group training group (86.2%), followed by the individual training group (70.0%). At the sixth week and fourth month follow-ups after birth, the highest rates were in the individual training group (93.3% and 63.3%, respectively). Inadequate milk supply, which was among the reasons for giving baby food other than breast milk, ranked first in the control group in the first week (83.3%), sixth week (42.1%) and fourth month (42.3%) after birth. Among the other reasons were the baby’s reluctance to suck the breast, breast problems, the working status of the mother, and attempts to introduce the baby to the taste of other foods. These were addressed as “others” in the table. As shown in Table 3, all participating mothers experienced breast problems in the first week after birth. The highest rates were in the individual training and control groups during the first week and in the control group during the sixth week (40.0%). The breast problem with the highest rate in the first week was cracked nipples.

Table 4 shows the comparison of the mothers in terms of the mean scores they obtained from the BSES during pregnancy and in the first week, sixth week and fourth month after birth. There were no significant differences between the mean scores obtained from the BSES and its subscales during pregnancy and at the first week follow-up after birth in all three groups (p> 0.05). However, in the sixth week postpartum, while there were differences between the groups in terms of their mean scores for the overall BSES (p<0.05), there were no differences between the groups in terms of their mean scores for the Intrapersonal Thoughts Subscale (p>0.05). In the sixth week after birth, the group training group obtained the highest mean scores from the BSES and its Intrapersonal Thoughts Subscale. For the comparison of the mean scores obtained from the BSES and its subscales in the fourth month postpartum, there were differences between the groups (p <0.05). The participants in the individual training group obtained higher scores than the participants in the other two groups. The intragroup comparisons of the mean scores obtained from the BSES and its subscales during the postpartum period yielded significant differences in all three groups (p<0.05). The total scores for the BSES were the highest in the individual training group in the fourth month, in the group training group in the sixth week and in the control group in the fourth month.

**Table 4:** Comparison of the mothers in terms of the mean scores they obtained from the BSES *Kruskal–Wallis ** K-Related Samples Breastfeeding self-efficacy scale (BSES) scores were calculated during pregnancy, and the first week, sixth week, and fourth month after birth.

	Pregnancy (before education)	Postpartum first Week	Postpartum sixth week	Postpartum fourth month	p*
Administration of the BSES	Mean ± SD	Mean ± SD	Mean ± SD	Mean ± SD	
BSES total score					
Individual education group	134.66±12.22	151.4±4.77	144.26±13.98	158.73±5.94	0.000
Group education group	135.00±16.61	152.5±9.35	153.75±7.44	151.10±6.11	0.000
Control group	130.83±13.04	124.0±13.16	129.66±12.57	147.93±15.19	0.000
p**	0.80	0.12	0.01	0.00	
Intrapersonal thoughts subscale					
Individual education group	82.46±6.71	88.03±4.25	83.86±9.60	92.43±2.96	0.000
Group education group	82.89±9.19	88.27±6.44	90.79±3.60	88.65±2.99	0.000
Control group	80.40±8.35	72.46±9.94	74.60±8.62	87.03±1.17	0.000
p	0.778	0.361	0.002	0.000	
Technique subscale					
Individual education group	52.20±7.31	63.36±2.79	60.40±7.41	66.56±4.04	0.000
Group education group	52.10±8.94	63.96±3.69	62.96±4.65	62.48±4.31	0.000
Control group	50.43±6.37	51.53±8.17	55.06±6.11	62.00±5.47	0.000
p	0.700	0.423	0.269	0.001	

The comparison of the participating mothers in terms of the mean scores they obtained from the IIFAS during pregnancy and in the first week, sixth week, and fourth month after birth demonstrated (Table 5) that there were no significant differences between the mothers in terms of the scores they obtained during pregnancy (p>0.005). Intergroup comparisons revealed significant differences between the groups in terms of the scores they obtained from the IIFAS during pregnancy and in the first week, sixth week and fourth month after birth (p<0.005).

**Table 5:** Comparison of the mothers in terms of the mean scores they obtained from the IIFAS *Kruskal–Wallis  ** K-Related Samples The Iowa Infant Feeding Attitude Scale (IIFAS) scores were calculated during pregnancy, and the first week, sixth week, and fourth month after birth.

	Pregnancy (before education)	Postpartum first week	Postpartum sixth week	Postpartum fourth month	p*
Administration of the IIFAS to the mothers	Mean ± SD	Mean ± SD	Mean ± SD	Mean ± SD	
Individual education group	63.76±5.17	68.8±3.86	74.10±3.28	73.20±3.51	0.000
Group education group	61.16±5.81	71.20±4.01	72.65±5.14	74.75±2.94	0.000
Control group	61.46±5.94	67.60±3.67	66.70±4.91	69.0±4.29	0.000
p**	0.151	0.006	0.000	0.000	
Administration of the IIFAS to the fathers					
Individual education group	64.03±8.25	66.93±16.00	67.96±17.50	66.96±18.00	0.000
Group education group	60.72±3.00	73.44±3.50	73.79±4.00	65.72±19.00	0.000
Control group	65.24±8.00	63.17±17.00	62.13±12.00	61.03±14.00	0.000
p	0.003	0.000	0.000	0.000	

According to the comparison of the participating fathers’ mean scores obtained from the IIFAS during their wives’ pregnancy and in the first week, sixth week and fourth month after birth, there were no significant differences in terms of the scores they obtained from the IIFAS at these time periods (p>0.005). There were statistically significant differences for both mothers and fathers between the scores they obtained at four different measurements (p<0.005).

## Discussion

This interventional study examined the effect of education on prospective mothers' and fathers' BSE and attitudes in the postpartum period. The prospective mothers and fathers were assigned into three groups: an individual training group, a group training group, and a control group. The sociodemographic characteristics of the mothers and fathers included in the study were similar.

The analysis of how the mothers fed their babies in the first week after birth demonstrated that EBF rates were high in all three groups. The highest rate of EBF in the sixth week was observed in the individual training group, followed by the group training group. In the fourth month, while the rate of EBF was still high in the mothers in the individual training and group training groups, it declined in the control group mothers. In Henshaw et al.’s study (2015), postnatal EBF rates were 75.55% on the second day, 61.76% in the sixth week and 38.18% in the sixth month [28]. In several interventional studies, EBF rates were higher in the participants receiving education [29,30]. These results are consistent with our results.

Nipple tenderness and pain are common in mothers who are new to BF in the first weeks after birth. In the present study, cracked nipples were observed in all three groups during the first week postpartum interviews. The presence of cracked nipples decreased in the sixth week and disappeared in the fourth month in all three groups. The study of Buck et al., conducted to investigate the presence of pain, damage, and vasospasm in nipples during the eight weeks after birth, found that 79% of the mothers had breast pain and 58% had nipple cracks, although they gave birth in a baby-friendly hospital [31]. Eksioglu et al. [29] used three different training techniques to heal nipple cracks, finding that the frequency of cracked nipples in the three groups ranged between 20% and 63.3% in the second week. At the end of the first month, although the incidence of cracked nipples dropped to 30% in the control group and to 10% in the education groups, the mothers still had cracked nipples. The literature review revealed that one of the most serious difficulties experienced by mothers within the first one or two weeks of BF in the postpartum period was breast problems. Of these problems, the leading ones were nipple pain and cracks [31]. In our study, despite giving education to the mothers in the individual and group training groups because they were first-time mothers, they suffered trauma and nipple cracks due to the baby's sucking. The results of other studies are consistent with our results.

The results of the present study confirmed the first hypothesis of the study: “The BF education method affects mothers' BSE.” Perceived BSE refers to the sufficiency that the mother feels about BF. The mother’s self-efficacy perception regarding breastfeeding may be related to difficulties she previously experienced in different situations [24]. In the present study, according to the measurements performed in the first week postpartum, the mothers in the individual and group training groups obtained higher mean scores from the BSES than did the mothers in the control group. In the sixth week’s measurements, the mothers in the group training group obtained the highest mean scores and the mean scores of the mothers in the control group increased, but there was a seven-point decrease in the mothers in the individual training group. However, in the fourth month’s measurements, the mean scores of the mothers in the individual training group were significantly higher than those of the mothers in the group training and control groups. Another situation supported by these findings was that EBF rates were high in the mothers who had high BSE scores. Several studies have demonstrated that antenatal BF interventions increase BSE in the postpartum period [30,32-35]. The findings of our study regarding the BF education given both to the mothers and fathers in the antenatal period are consistent with the findings in the literature, indicating that education given in the antenatal period increases the level of perceived BSE. In their study (2017), Park et al. also investigated the effect of BF education given to couples on BSE, BF satisfaction, and spousal support. They found that BF education given to couples increased BSE, BF satisfaction and spousal support [36]. Yurtsal et al. (2015) investigated the effect of antenatal BF education given to the mothers and their husbands together regarding the duration of BF and maternal and paternal attachment, determining that the EBF rate was 50.4 times higher in the mothers in the intervention group than in the mothers in the control group [37]. Aguirre et al. (2018) stated that the effect of BF education given in the antenatal period on BSE might change over time. In their study, there was no significant difference between the groups before education and in the first days after birth, but there was a significant difference between the groups in the sixth week, third month, and sixth month after birth [38]. The results of our study are consistent with those of Aguirre et al.’s study. While there was no difference between the groups in terms of their BSE scores obtained before the education and in the first week after birth, there was a difference between the groups in the sixth week and fourth month measurements.

Receiving BF education and maintaining BF behavior are thought to be effective in the development of attitude because attitude develops as the knowledge is reinforced and the behavior is repeated [39]. Infant feeding attitude is important because it mirrors the decision made by mothers during pregnancy and the postpartum period regarding their choice of feeding method: breastfeeding or bottle feeding. Therefore, the second hypothesis of our study: “BF education method affects mothers’ BF attitudes” is confirmed. Intergroup comparisons regarding the mean scores obtained from the IIFAS in the first week, sixth week and fourth month after birth demonstrated significant differences between the groups (p<0.05). The present study observed that the mothers in the control group also maintained BF behaviors, although they were not given structured education. In Eksioglu et al.’s study conducted in Turkey in 2016, the mean scores the participants obtained from the IIFAS in the sixth week after birth were higher than those obtained in the first weeks after birth [27]. In an intervention study conducted in Greece, there was a difference between education and control groups. While there was a significant increase in the education group’s IIFAS score after the education, the control group’s IIFAS score increased only by 0.03 [35]. Abuidhail et al. conducted a study in Jordan in 2019, investigating the effect of web-based prenatal BF education on the participants’ BF attitudes in the third trimester and found no difference between the participants in the intervention and control groups in terms of their attitudes toward BF. They also determined that the pregnant women participating in their study displayed positive attitudes toward neither BF nor bottle feeding [40]. The results of Abuidhail et al.’s study were different from the results of our study. In our study, thanks to the education intervention, the participating mothers’ attitudes changed over the process. This difference probably stemmed from the fact that the participants were of different cultural backgrounds, Abuidhail et al.’s study was conducted in the third trimester and the techniques used were different. This is because BF is a behavior affected by the culture. In our study, the IIFAS scores of the mothers who received individual or group training in the antenatal period were higher than those of the mothers in the control group, revealing the positive effect of the BF education given in the antenatal period. Although the participants in the control group did not receive any education, their scores also increased. This suggests that ensuring the continuity of BF behavior, although partly, affected attitude development.

Another factor that plays an important role in BF is husbands' supportive attitudes toward BF. The results of the present study confirmed the third hypothesis of the study: “The BF education method affects fathers' BF attitudes”. The husband's display of positive attitudes toward BF and supportive behaviors toward the mother is the main factor affecting the mother's maintenance of BF [41]. There were differences between the fathers included in the study in terms of the mean scores they obtained from the IIFAS at the first follow-up before education and at the first week, sixth week, and fourth month follow-ups after birth (p<0.05). The difference between the groups in terms of BF attitudes before the education could be attributed to husbands developing a BF attitude in the antenatal period. In Abbass-Dick et al.’s BF intervention study (2015), there was no difference between the groups in terms of mean IIFAS scores obtained during the pre-education period and at the sixth week follow-up. The increase in the mean IIFAS score obtained in the sixth week of follow-up was 1.3 points in the intervention group and 0.1 points in the control group [19]. In Mitchell-Box et al.’s study [42], the mean IIFAS score obtained by the fathers of the babies who were EBF in the hospital was 65.9 ± 7.2, that of the fathers of the babies who were only formula fed was 51.8 ± 1.7 and that of the fathers of the babies who were both breastfed and formula fed was 58.7 ± 7.6.

In the same study, when the fathers’ attitudes were reassessed in the first few weeks, the mean IIFAS score obtained by the fathers of the babies who were EBF was higher than that of the fathers of the babies who were only formula fed and that of the fathers of the babies who were both breastfed and formula fed.

In our study, although there was a statistically significant difference between the mean IIFAS scores of the fathers in each group, the mean scores of the fathers in the control group decreased over time. The mean IIFAS score of the fathers in the individual training group did not change significantly from pregnancy to the fourth month after birth. On the other hand, the mean IIFAS score of the fathers in the group training group, which increased from the first follow-up to the sixth week of follow-up after birth, was similar to the mean scores of the other two groups at the fourth month of follow-up after birth. These results indicate that the education given to the fathers in a group contributed to their developing favorable attitudes toward BF. Because the fathers in the control group did not receive any education, their BF attitude development was not sufficient. In the literature, there is a limited number of studies comparing the relationship between BF education and fathers' attitudes toward BF. In Abbas-Dick’s study [19], there was a minor change in the BF attitudes of the fathers in the control group whose wives were subjected to routine hospital interventions over the six weeks after birth. The results of our study are consistent with those of Abbas-Dick’s study. In our study, education was provided through two methods (individual and group training) and the group training was found to be more effective in fathers’ developing BF attitudes. Daniele et al. (2018) stated that group sessions should be provided to encourage men to think critically about patriarchal norms [43], which supports the results of our study and indicates that group training affected attitude development in fathers more.

## Conclusions

The results of the present study conducted to investigate the effect of individual and group BF education provided to mothers and fathers on their BSE and attitudes in the postpartum period confirmed the first hypothesis of the study: “The BF education method affects mothers' BSE.” The results also confirmed the second hypothesis "The BF education method affects mothers’ BF attitudes" and the third hypothesis “The BF education method affects fathers' BF attitudes”. The analysis of all the results indicates that BF education given to parents in the antenatal period increases their BSE and fosters their positive attitude toward BF. However, to determine whether individual or group training contributes to the development of BSE and attitudes, further research is needed. BSE and attitudes improve over the postpartum period. Therefore, positive changes are expected in behaviors and attitudes toward BF as the postpartum period progresses because the postpartum process is a recovery period in which the mother renews herself and adapts to changes in the family life cycle.
